# Incidence, Diagnosis and Repair of a Diaphragmatic Hernia Following Hepatic Surgery: A Single Center Analysis of 3107 Consecutive Liver Resections

**DOI:** 10.3390/jcm10051011

**Published:** 2021-03-02

**Authors:** Jonas Raakow, Ioannis-Fivos Megas, Moritz Schmelzle, Wenzel Schoening, Georg Lurje, Matthias Biebl, Johann Pratschke, Panagiotis Fikatas

**Affiliations:** Department of Surgery, Campus Charité Mitte and Campus Virchow-Klinikum, Charité—Universitätsmedizin, Corporate Member of Freie Universität Berlin, Humboldt-Universität zu Berlin and Berlin Institute of Health, Augustenburger Platz 1, 13353 Berlin, Germany; jonas.raakow@charite.de (J.R.); fivos.megas@gmail.com (I.-F.M.); moritz.schmelzle@charite.de (M.S.); wenzel.schoening@charite.de (W.S.); georg.lurje@charite.de (G.L.); matthias.biebl@charite.de (M.B.); johann.pratschke@charite.de (J.P.)

**Keywords:** diaphragmatic hernia, liver resection, hernia repair, mesh, enterothorax

## Abstract

Diaphragmatic hernia (DH) after a liver resection (LR) is an uncommon but potentially severe complication. In this retrospective study, we aim to share our experience with DH in our hepatic surgery center. We retrospectively analyzed 3107 patients who underwent a liver resection between January 2012 and September 2019. The diagnosis of DH was based on clinical examination and radiological imaging and confirmed by intraoperative findings during surgical repair. Five out of 3107 (0.16%) patients after LR developed DH. Especially, all five DH patients had a major right-sided LR before (*n* = 716, 0.7%). The mean time interval between initial LR and occurrence of DH was 30 months (range 15 to 44 months). DH exclusively occurred after a right or extended right hepatectomy. Two patients underwent emergency surgery, three were asymptomatic, and DH was diagnosed in follow-up imaging. Three of these five treated patients (60%) developed DH recurrence: two of three (67%) patients after suture repair alone and the only patient after suture repair in combination with an absorbable mesh. The patient who was treated with a composite mesh implant did not show any signs of DH recurrence after 52 months of follow-up. In patients who develop DH after liver surgery, a mesh augmentation with nonresorbable material is generally recommended. In order to diagnose these patients in an early state, we recommend that special attention be paid and a prompt and targeted diagnostic examination of patients with abdominal complaints after right-sided liver resections take place.

## 1. Introduction

An acquired diaphragmatic hernia (DH) is a rare condition, occurring either due to trauma or after major liver resections. When resulting from a blunt or penetrating trauma, they are usually located on the left side because on the right side, the liver covers the diaphragm and thus protects it from injury [1,2]. The incidence of DH following trauma is 3.4% for blunt trauma and 2.1% for penetrating trauma [3]. Hence, a right-sided hernia can occur after liver resections, especially following a right-sided extended hepatectomy [4,5,6,7,8,9]. There are also some cases of DH after living donor transplantation described in the literature [10]. Other iatrogenic diaphragmatic hernias have been reported following esophagogastrectomies, hiatus hernia repairs, radiofrequency ablation of liver tumors or due to endometriosis [11,12,13,14]. Altogether, it remains a rare postoperative complication after hepatic resections [15]. Clinical appearance ranges from completely asymptomatic to bowel obstruction or even bowel perforation with severe peritonitis [16]. In this study, we present a cohort of patients with a right-sided diaphragmatic hernia following liver resection who underwent conventional or laparoscopic hernia repair. The aim was to present patient-related data, the surgical therapy and the postoperative course of this uncommon but potentially severe complication after major liver resection.

## 2. Materials and Methods

The Department of Surgery Campus Charité Mitte/Campus Virchow-Klinikum, Charité–Universitätsmedizin Berlin, Germany is a high-volume hepatobiliary center with an average of more than 400 cases per year [17]. We retrospectively analyzed 3017 patients who underwent liver surgery between January 2012 and September 2019. Patient’s demographics, operative details and circumstances as well as time to diaphragmatic hernia occurrence and the surgical outcome were examined. The study was conducted in accordance with the requirements of the Institutional Review Board of the Charite–Universitätsmedizin Berlin, the current version of the Declaration of Helsinki, and the guidelines for good clinical practice.

The initial liver resection of all DH cases was performed using an open approach. Liver resection was carried out in accordance with common clinical standards. The right liver lobe was fully mobilized before parenchymal transection and was performed by cutting of the right triangular ligament and the right diaphragmatic adhesions to the liver. This step was performed with either a monopolar cautery or scissors. Hemostasis on the right side of the diaphragm was achieved with a bipolar clamp or an infrared coagulator.

Diagnosis of DH was either an accidental finding, a finding during planned follow-up examinations or found in an emergency consultation. Contrast-enhanced computed tomography (CT) or magnetic resonance imaging (MRI) was used to prove the diagnosis.

For DH repair, either an open (*n* = 4, 80%) or a laparoscopic approach (*n* = 1, 20%) was used, and the defect was treated with a primary repair using a nonabsorbable suture only or in combination with placement of a prosthetic mesh implant (Table 1).

Statistical analysis in this study was carried out using the Statistical Package for Social Sciences software (IBM SPSS). For categorical variables analysis, Fisher’s exact test was applied. The level of significance was set to *p* < 0.05, and *p*-values are given for two-sided testing. Analyses were performed using SPSS Statistics 25 (IBM Corp., Armonk, NY, USA).

## 3. Results

**Figure 1:**
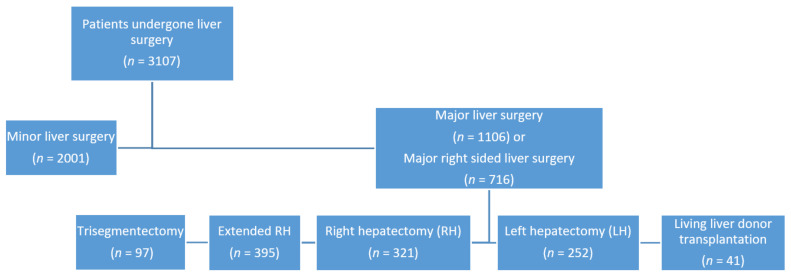
Flowchart of the 3107 cases of liver resections at Department of Surgery at Charité – Universitätsmedizin Berlin from January 2012 to September 2019.

Out of 3017 identified liver resections from a prospectively maintained database from January 2012 to September 2019 (*n* = 3107), five (0.16%) patients developed postoperative DH (Figure 1).

DH only occurred in patients who underwent major right-sided liver resections (anatomical right-sided or extended right-sided hepatectomy). During this period, 716 elective major right-sided liver resections were performed for benign, malignant tumors and infective remnants.

The incidence following major right-sided liver resection was 0.7% (*n* = 5). Two patients received liver resections because of colorectal metastasis (40%), one because of cholangiocarcinoma (20%) and one because of hepatocellular carcinoma (20%). Only one hepatectomy was performed without prior tumor diagnosis: one severe case of cholecystitis with huge liver abscess (20%). In less extensive surgeries such as wedge resections, segmental or anatomical left resection, there was no incidence of DH. No case of DH after living liver donation was found at our center.

The average patients’ age at time of DH diagnosis was 57.5 years. The median BMI was 27.9 kg/cm^2^. Only one of them was male (20%). On average, 30 months passed between the operation and the clinical occurrence of the hernia, ranging between 15 and 44 months. Three patients (60%) with DH presented with a symptomatic condition (abdominal pain, shortness of breath or bowel obstruction symptoms). Only two (40%) DH patients received emergency surgical treatment, one of them because of an ileus and the other one because of peritonitis due to a jejunal perforation, needing concomitant bowel resection. In the remaining three patients (60%), DH were random findings in the course of a routine follow-up. The diagnosis was mainly made with CT. In one case, an additional MRI was performed because of hepatocellular carcinoma (HCC) recurrence in order to obtain the most possible information about tumor progress. One of the patients received laparoscopic repair (20%), and four (80%) were carried out with an open approach because of intra-abdominal adhesions. Three of the patients were treated with a nonabsorbable suture and two received a prosthetic mesh implant (BioA^®^, Gore, 7 × 10 cm and Parietex Composite^®^, Medtronic, 20 × 15 cm). In all patients, a pleural drainage (≥20 Ch) was placed.

The perioperative period regarding DH was uneventful in all patients. Three patients (60%) showed a recurrence in the follow-up. One patient had ileus symptoms one year after primary DH repair, and was then treated with implantation of a mesh (Bio-A^®^, Gore). The other two recurrent patients were diagnosed in routine follow-up radiological examinations one year after DH repair. Surgical repair of DH recurrence had not been performed yet.

We analyzed the recurrence rate after DH repair. For this reason, the patients were divided into two groups: group one, with primary repair only or in combination with a resorbable mesh, and group two, with nonresorbable mesh augmentation. No significant difference was found (*p* = 0.4).

**Figure 2:**
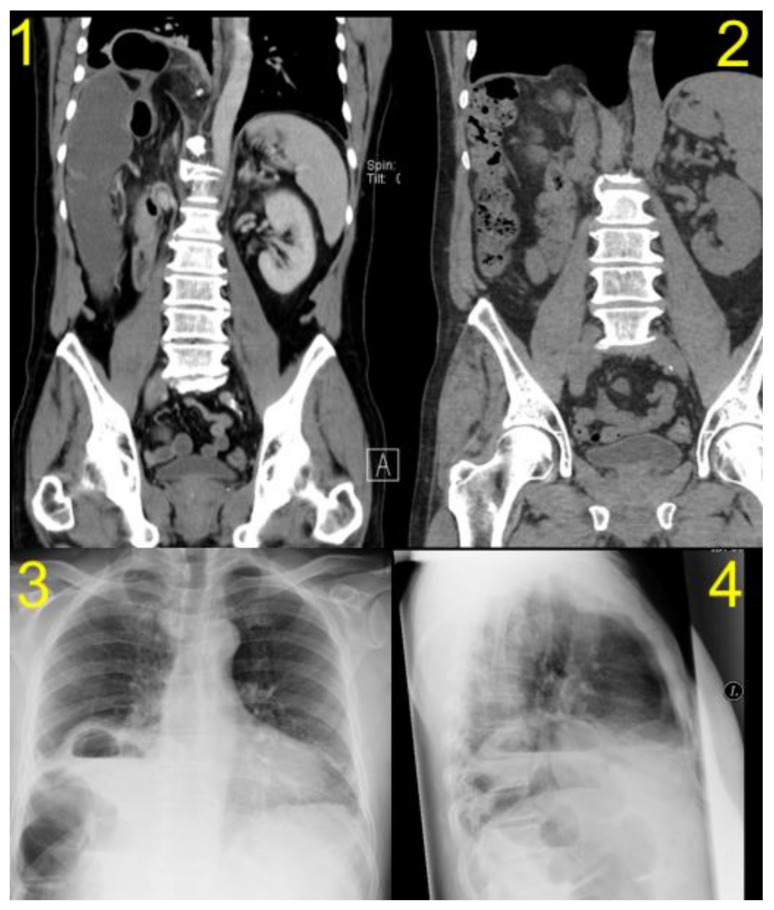
Pre- and postoperative CT scans of one of the patients with diaphragmatic hernia (DH) after right liver resection: (**1**) preoperative computed tomography (CT) scan, (**2**) follow-up CT scan showing the recurrence of DH after primary suture repair, (**3**) and (**4**) chest X-ray images before DH repair.

The characteristics of the patients are shown in summary in Table 1. Preoperative and recurrence CT scans of one DH patient are shown in Figure 2.

## 4. Discussion

Diaphragmatic hernia constitutes a rare but potentially severe complication following major liver resection. As a high-volume hepatobiliary center, we seek to stress the role of DH, its importance in the differential diagnoses of right upper abdominal pain and ileus symptoms in patients who underwent liver resection. A variety of additional presenting symptoms are described, such as respiratory distress, abdominal distension, constipation, spasmodic hiccup with chronic right upper abdominal pain and acute or chronic bowel obstruction symptoms being the common ground [4,5,15,16,18,19].

The reasons for the occurrence of DH after liver surgery have not yet been sufficiently clarified. The resection or opening of parts of the diaphragm in primary surgery as well as the initial tumor size are suggested as possible risk factors in the literature. This observation was also made in our cohort, as DH occurred after liver resections due to larger lesions, such as multifocal tumors, larger tumor diameter or an abscess needing extensive parenchymal resection. Additionally, cautery-related thermal injury is most often mentioned in this context. Nevertheless, the etiology seems to be multifactorial [4,5,7,20,21,22].

In our cases, the diagnosis was made based on CT scan findings. In general, two radiological methods could be used to confirm the diagnosis. As CT scan is a part of most cancer follow-up examinations and at the same time the most effective procedure, it should take center stage in routine diagnostics, while sonography, being even easier to perform, could be used as first-line in an emergency situation or in outpatient clinic consultations by experienced examiners [23]. Although in synopsis of the studies it is shown that the sensitivity of chest X-ray (CXR) for diaphragmatic injuries varies between 40% and 81%, with some studies also speaking of an error rate of 40%, in our opinion, CXR is not a meaningful method, while ultrasound is superior, being proven in several studies as a reliable method of diagnosing diaphragmatic hernias [24,25,26,27,28,29,30].

In the literature, the recommended follow-up is 24 months [15]. The median time interval between the initial liver resection in our study was 30 months, longer than the 19 months described in the literature [15]. Based on our experience with the occurrence of a DH 44 months after LH, we recommend at least 48 months of follow-up. Oh et al. recommended that clinicians and radiologists should not overlook DH after living-donor right hepatectomy [19]. We would like to endorse the thesis and propose, in addition to the standardized follow-up for carcinomas, a targeted diagnostic examination of patients with abdominal complaints after right-sided liver resections.

Our retrospective study showed a lower incidence of diaphragmatic hernias after liver resection than previously reported in the literature. The figures described in the literature range from 1% to 2.3% and are thus higher than the 0.16% and 0.7% reported in our study [4,5,15]. To this day, the number of publications on this topic is low. Our department carries out a high number of liver resections every year, so we believe that a lower incidence of DH is a logical consequence of this expertise. More specifically, when mobilizing the right liver lobe, care is taken not to injure the diaphragm. For this purpose, only the ligamentum triangulare dextrum is dissected. This also includes avoiding severe bleeding of the diaphragm during dissection, which could lead to extensive thermocoagulation and secondary damage to the diaphragm [15].

Regarding the surgical treatment of DH, some authors recommend that a hernia smaller than 10 cm should be treated with a primary suture and a hernia larger than 10 cm with mesh [4,5,7]. Based on our experience, we do not recommend primary suture repair but prefer mesh augmentation, because the treatment of a small hernia without mesh led to a recurrence, which had negative influence on the clinical outcome and morbidity of the patient (Table 1). This opinion has already been reflected in literature for DH after a blunt trauma [31].

In our opinion, prompt treatment of the diaphragmatic hernia and augmentation by means of mesh are indispensable to avoid possible complications and thus reduce morbidity [4,5]. In this context, it should also be mentioned that early diagnosed DH defects are small or moderate in size and could therefore be repaired more easily [5].

Although our analysis did not show statistical significance, the numbers are quite obvious—80% of the patients not treated with a nonresorbable mesh showed a recurrence of DH. Therefore, we recommend an augmentation with a nonresorbable and intraperitoneal suitable mesh for all DH after LR because in our opinion, this is the best method to avoid recurrence. Right now, no data exist about DH and repair with long-term resorbable meshes.

In this study, only abdominal approaches were chosen. If technically possible, this should always be preferred to the thoracic approach, in order to avoid a two-cavity procedure [5,7,8]. On the other hand, Tabrizian et al. suggested that a thoracic approach is useful for recurrence of DH after repair by an abdominal approach [5].

Furthermore, a laparoscopic approach is superior to an open approach with regard to convalescence, especially with regard to postoperative pain and mobilization. The postoperative complication rate is lower at experienced centers after laparoscopic liver resection than after conventional open surgery [17]. Further studies may also verify this for a diaphragmatic hernia after a liver resection. Finally, advances in laparoscopic and robotic surgery will show in the future whether the incidence of DH after liver resection will change or can also be reduced by, for example, a more detailed dissection [17,32,33].

## 5. Conclusions

Due to the severity of complications in patients with diaphragmatic hernia, we recommend (if possible) defect closure by nonresorbable suture with mesh augmentation of every diaphragmatic hernia after liver resection. Mesh repair can be performed laparoscopically in IPOM technique or as open mesh implantation. We suggest that this maximal reinforcement of the diaphragmatic defect reduces the risk of recurrence and consecutive morbidity. Hernia repair under elective conditions would improve the clinical outcome of patients compared to emergency surgery. Therefore, we recommend, in addition to the standardized follow-up of carcinomas, a targeted diagnostic examination of patients with abdominal complaints after right-sided liver resections, especially in cases of extensive preparation or dissection of the diaphragm, in order to diagnose a DH at the earliest possible state and certainly not in an emergency situation. Special attention should be given to the few noncancer patients after right-sided liver resections in case of hernia-related symptoms, since they do not have standardized follow-up.

## Figures and Tables

**Table 1:** Characteristics of the patients with diaphragmatic hernia after liver surgery.

	Patient 1	Patient 2	Patient 3	Patient 4	Patient 5
Age	54	56	58	49	69
Gender	m	f	f	f	f
BMI	26.1 kg/cm^2^	27.9 kg/cm^2^	35.7 kg/cm^2^	20 kg/cm^2^	29.4 kg/cm^2^
Etiology of LR	CRLM	CCC	CRLM	HCC	cholecystitis
Procedure	ext. right hepatectomy	Right hepatectomy	Right hepatectomy	ext. right hepatectomy	ext. right hepatectomy
Open or laparoscopic approach	open	open	open	open	open
Size Of Tumor	multiple leasons	10 cm	multiple leasons	18 cm	parenchymal abscess
Resection Of Diahpragm in the Course Of The LR	no	no	no	no	no
DH Occurence: Time after Liver Resection	21 months	15 months	34 months	44 months	36 months
Symptoms/Reason of Presentation	ileus	shortness of breath	colon stenosis during coloscopy	HCC recurrence in Follow-up, asymptomatic in respect of DH	enterothorax with jejunal perforation and peritonitis
Diagnostic Study	CT	CT	CT	CT/MRI	CT
Herniated organ	right colon flexure, omentum	colon and small bowell	colon and omentum majus	colon	colon
Side of hernia	right-sided	right-sided	right-sided	right-sided	right-sided
Size of Hernia	4 cm	<5 cm	4 cm	5 cm	7 cm
Elective/Emergent	emergent	elective	elective	elective	emergent/elective
DH Repair Approach	Open	Open	Lap.	Open	Open
DH Procedure	primary repair	repair with mesh BioA 10 × 7 cm	repair with composite IPOM	primary repair	primary repair/BioA Mesh at recurrence
Chest Drain During DH Repair	yes	yes	yes	yes	yes
Complications after DH Repair	recurrence	recurrence	none	none with regard to DH	recurrence
Hospital Stay after DH Repair	9 days	8 days	5 days	15 days	21 days
Follow up after DH Repair	36 months	41 months	52 months	14 months	62 months
Recurrence after DH Repair	yes	yes	no	no	yes
Time After DH Repair Till Diagnosis of Recurrence	12 months	12 months	-	-	22 months
